# Characteristics of Lignin from Flax Shives as Affected by Extraction Conditions

**DOI:** 10.3390/ijms11104035

**Published:** 2010-10-20

**Authors:** Kelly Ross, Giuseppe Mazza

**Affiliations:** Pacific Agri-Food Research Center, Agriculture and Agri-Food Canada, 4200 Highway 97, Summerland, BC, V0H 1Z0, Canada; E-Mail: giuseppe.mazza@agr.gc.ca

**Keywords:** flax shives, biomass, lignin, extraction, pressurized low polarity water, PLPW, subcritical water, nitrobenzene oxidation, UV spectroscopy, Pyrolysis-GC-MS

## Abstract

Lignin, a polyphenolic molecule, is a major constituent of flax shives. This polyphenolic molecular structure renders lignin a potential source of a variety of commercially viable products such as fine chemicals. This work compares the performance of different lignin isolation methods. Lignin from flax shive was isolated using both conventional alkaline extraction method and a novel experimental pressurized low polarity water (PLPW) extraction process. The lignin yields and chemical composition of the lignin fractions were determined. The conventional alkali treatment with 1.25 M NaOH, heated at 80 °C for 5 h, extracted 92 g lignin per kg flax shives, while lignin yields from the PLPW extracts ranged from 27 to 241 g lignin per kg flax shives. The purity and monomeric composition of the lignins obtained from the different extraction conditions was assessed via UV spectroscopy and alkaline nitrobenzene oxidation. Lignin obtained from conventional alkali treatment with 1.25 M NaOH, heated at 80 °C for 5 h was of low purity and exhibited the lowest yields of nitrobenzene oxidation products. With respect to alkali assisted PLPW extractions, temperature created an opposing effect on lignin yield and nitrobenzene oxidation products. More lignin was extracted as temperature increased, yet the yield of nitrobenzene oxidation products decreased. The low yield of nitrobenzene oxidation products may be attributed to either the formation of condensed structures or the selective dissolution of condensed structures of lignin during the pressurized alkaline high temperature treatment. Analytical pyrolysis, using pyroprobe GC-MS, was used to investigate the molecular composition of the lignin samples. The total yield of pyrolysis lignin products was 13.3, 64.7, and 30.5% for the 1.25 M NaOH extracted lignin, alkaline assisted PLPW extracted lignin, and the unprocessed flax shives, respectively. Key lignin derived compounds such as guaiacol, 4-vinyl guaiacol, 4-methyl guaiacol, syringol, eugenol, isoeugenol, catechol, homocatechol, and vanillin were detected in all of the samples.

## 1. Introduction

The phenomenon of global warming and its impact on the environment has called attention to the use of more environmentally friendly processes and the use of agricultural waste as a lignocellulosic biomass resource to ensure environmental, social, and economic sustainability [1]. Flax shives are considered the waste product remaining after cellulose fiber removal from flax straw. The yield of shives is 2.5 tons for every one ton of fiber produced [2]. The large amounts of agri-based lignocellulosic biomass produced highlights the need for positioning a material conventionally deemed a waste product of agricultural production as a co-product, and subsequently as bioresource.

Flax shives possess high lignin (∼24%), cellulose (∼53%) and hemicellulose (∼24%) contents [3,4]. With regards to textiles and pulp and paper products, lignin has long been considered detrimental to quality because the lignin is either dark in color or, if whitened by bleaching, it tends to darken due to auto oxidation and/or photo oxidation during aging [5]. However, it is also the chemical composition of lignin that makes it an appealing resource for high value chemicals. Structurally, lignin is a phenolic high molecular weight (600–1500 kD) polymer that serves several functions in vascular land plants [5–7]. Chemically, lignin is a heterogeneous polymer composed of phenolic compounds, namely, coniferyl alcohol, sinapyl alcohol, p-coumaryl alcohol that lead to guaiacyl (G), syringyl (S), and p-hydroxyphenyl (H) propane type units [6]. These phenylpropane monomers are linked by several types of carbon-oxygen (ether) and carbon-carbon bonds. Condensed lignins contain more biphenyl, biphenyl ethers linkages (*i.e.*, condensed linkages) while non-condensed lignins contain more alkyl-aryl ether (β-*O*-4, α-*O*-4) linkages (*i.e*., non-condensed linkages) [5,8]. The heterogeneity exhibited by lignin is due to variations in composition, size, degree of crosslinking, and functional groups [8,9]. Lignin composition depends upon genetic origin, plant tissue type, and the extraction method used to obtain the lignin from lignocellulosic biomass [9]. In monocotyledonous Angiospermae, other than grasses (Gramineae) and dicotyledonous Angiospermae including both plants such as flax and hemp and hardwoods, lignin is mainly derived from coniferyl and sinapyl alcohols in various quantities. In monocotyledonous Angiospermae grasses such as cereal straws, the lignins are composed of guaiacylsyringyl- type lignin cores and 4-hydroxycinnamic acid type peripheral groups [4,9]. In Gymnospermae plants (*i.e.*, softwoods), lignin structural elements are predominantly derived from more than 95% coniferyl alcohol. It is the large amount of functionalized aromatic structures present in lignin which makes it an attractive raw material for aromatic compounds [10]. Depolymerization of lignin into base chemicals is one of the key areas for valorization of lignin [11].

Compositional analysis of lignin is typically performed by chemical or thermal degradation of the lignin macromolecule into small compounds which are separated by means of chromatographic techniques [12]. Key chemical methods that have been used to characterize lignin include thioacidolysis and nitrobenzene oxidation. Thioacidolysis disrupts the noncondensed intermonomer linkages (alkyl-aryl ether) giving rise to thioether derivatives of the p-hydroxyphenyl (H), guaiacyl (G), and syringyl (S) lignin monomer units while nitrobenzene oxidation cuts the propane side chains giving rise to aldehydes of these monomers [5].

Analytical pyrolysis is another useful technique for lignin analysis [12]. Pyrolysis (Py) thermally degrades polymers into small fragments in the absence of oxygen with heat alone [13]. These components can then be separated with gas chromatography (GC) and identified with the aid of mass spectrometry (MS). Pyrolysis coupled with gas chromatography and mass spectroscopy (Py-GC-MS) can provide a finger print of the starting macromolecule and give information on the relative amount of its monomeric components [12,14]. Ralph and Hatfield [15] characterized the chemical differences in cell wall structural components of forage material using Py-GC-MS. Greenwood *et al*. [16] demonstrated the value of pyrolysis to study wood composition. Furthermore, pyrolysis is more than a useful analytical technique; it is also a technology that can be used for the thermal chemical conversion of lignin into high value base aromatic chemicals [17].

Valorization of lignin represents a great opportunity in terms of economic, environmental and societal sustainability; however the separation of lignin from cellulose and hemicellulose is very difficult. The cause for this has been suggested to be the strong bonds between lignin, hemicellulose, as well as the crystalline regions of cellulose. Processes traditionally used for fractionation of lignocellulosic biomass are acid hydrolysis, alkaline hydrolysis, ammonia treatments, and oxidation [18]. Further, Kim and Mazza [18] noted both economical and environmental concerns with regards to using conventional lignin extraction procedures. Citing efficiency and environmental concerns, they developed a pressurized low polarity water (PLPW) extraction regime to successfully extract and separate cellulose, hemicellulose and lignin from flax shives. PLPW extraction employs water heated to temperatures of between 100 and 374 °C and subjected to high pressure (<22 MPa), which keeps the water in the liquid state. Under these conditions the solubility of organic compounds is greatly enhanced as the water is much less polar, hence the name pressurized low polarity water. Nevertheless, various types and concentrations of alkali, as well as pretreatment time and temperature conditions, have been comprehensively studied in the extraction of wheat straw lignins [19,20]. Sun *et al*. [21] have demonstrated that straw and grass lignins can be isolated by mild alkaline treatments. Mild alkaline treatment does not cause significant chemical modification beyond hydrolysis of ester bonds between phenolic acids and lignin or polysaccharides [21]. Even so, any process used for removing lignin from the plant biomass changes their form and chemical makeup to some degree. This complicates lignin characterization and influences their functionality and utilization.

The main objectives of this work were to: (1) characterize flax lignin isolated via a conventional alkaline extraction procedure; (2) characterize flax lignin isolated via an environmentally benign process, PLPW; (3) provide a comparison of the characteristics of the flax lignins extracted with different methods; and (4) apply analytical pyrolysis to provide information on the chemical composition of the lignin samples.

## 2. Results and Discussion

### 2.1. Effect of Extraction Conditions on Lignin Yield, Purity and Composition

Table 1 shows the composition of the flax shives, namely, acid insoluble lignin, acid soluble lignin, cellulose, hemiceullulose, wax and ash content (g kg^−1^ flax shives, w/w dry basis). The amount of Klason lignin (acid insoluble lignin) present in the flax shives as determined by the NREL method [22] was 26% and the acid soluble lignin content was 1.3%. The total of these two lignin values, 27.3%, was used to calculate the extraction efficiency.

Table 2 shows the effect of conventional alkali treatment and pressurized low polarity water (PLPW) on the extraction of lignin from flax shives. Conventional alkali extraction with 1.25 M NaOH, at 80 °C for 5 h yielded 92 g kg^−1^ lignin on a flax shives basis extracting nearly 34% of the lignin present in the flax shives. Extraction temperature positively affected the amount of lignin obtained from alkali assisted PLPW processing as the highest lignin yield (241 g kg^−1^ lignin on a flax shives basis) was obtained with alkali assisted PLPW extraction with 0.47 M NaOH at 180 °C. This treatment extracted nearly 88% of the lignin present in the flax shives. More lignin was present in the 0.47 M NaOH-180 °C extract, 241 g kg^−1^, compared to the water-180 °C extract, 27 g kg^−1^. Notably, the extraction conditions of water-180 °C yielded less lignin than the 1.25 M NaOH, at 80 °C for 5 h extraction condition. These results demonstrate the effectiveness of alkali assisted pressurized low polarity water a method for extracting lignin from lignin-carbohydrate complexes. Delignification is achieved through cleavage of phenolic α-*O*-4-linkages, β-*O*-4-linkages, non-phenolic β-*O*-4-linkages, and carbon–carbon linkages in lignin and is therefore enhanced in an alkaline extraction medium [23]. Both the extracts from the water-180 °C and 0.47 M NaOH-180 °C PLPW extraction conditions displayed the highest carbohydrate contents of 311 and 302 g kg^−1^ and free phenolic acid contents of 3.9 and 4.7 g kg^−1^, respectively. Both pH and temperature have been shown to affect removal of carbohydrates from lignin-carbohydrate complexes. Increased removal of carbohydrates from flaxseed meal was achieved through the use of neutral and acidic extraction solvents [24] compared to alkaline extraction solvents, which is contradictory to our results with lignin-rich flax shives. However, extraction temperature has a positive effect on carbohydrate removal [24]. Presumably, higher temperatures promote the weakening of carbohydrate-lignin bonds and thereby enhance the dissolution of carbohydrate material into the extraction solvent [25]. Moreover the work of Ho *et al*. [24] reported that extraction temperature was the dominant factor affecting carbohydrate removal; the effect of pH of the extraction solvent was not significant compared to temperature. Our results are in agreement with these findings. With respect to extraction of phenolics, a positive effect of both increasing pH and increasing temperature has been reported [26], which is in agreement with our results.

**Figure 1:**
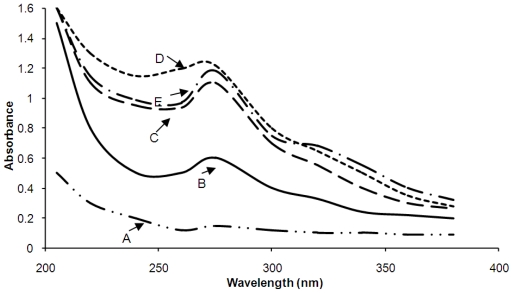
UV spectra of lignins isolated using: (**A**) conventional alkaline extraction with 1.25 M NaOH at 80 °C for 5 h; (**B**) alkaline assisted PLPW extraction with 0.47 M NaOH at 100 °C for 82 min; (**C**) alkaline assisted PLPW extraction with 0.47 M NaOH at 140 °C for 82 min; (**D**) alkaline assisted PLPW extraction with 0.47 M NaOH at 180 °C for 82 min; and (**E**) PLPW extraction with water at 180 °C for 82 min.

UV-visible light absorption measurements have been used to semi-quantitatively assess the purity of lignin samples [27–29]. The intensity of the absorbance is related to the level of lignin concentration and is proportional to the purity level of lignin. Therefore, a lower absorbance indicates the presence of non-lignin material such as carbohydrates [28]. Figure 1 presents the UV spectra of lignins isolated using: (A) conventional alkaline extraction with 1.25 M NaOH at 80 °C for 5 h; (B) alkaline assisted PLPW extraction with 0.47 M NaOH at 100 °C for 82 min; (C) alkaline assisted PLPW extraction with 0.47 M NaOH at 140 °C for 82 min; (D) alkaline assisted PLPW extraction with 0.47 M NaOH at 180 °C for 82 min; and (E) PLPW extraction with water at 180 °C for 82 min. In Figure 1, the maximum absorbance for all lignin samples occurred at 280 nm, which originated from the non-conjugated phenolic groups in the lignin [27,28]. Interestingly, as shown in the spectra, the highest absorbance values occurred in the lignin samples obtained from the pressurized 0.47 M NaOH-180 °C and water-180 °C extracts, suggesting that these were the most pure lignin preparations. Although these extracts contained the highest carbohydrate contents, the lignins present in the extracts were relatively pure (*i.e.*, minimal bound carbohydrate). Alternatively, the lowest absorbance values were observed in the lignin samples obtained from the 1.25 M NaOH-80 °C and 0.47 M NaOH-100 °C treatments and although the 1.25 M NaOH-80 °C treatment yielded 92 g lignin per kg flax shive, these lignin samples likely contained higher amounts of bound carbohydrate and were less pure. These results indicate that even though delignification is enhanced in an alkaline solvent the efficiency of alkali assisted PLPW is superior to conventional alkaline extraction. The pressurized solvent possesses decreased surface tension and viscosity, which allowed for enhanced extraction [30]. The improved extraction capability increases as temperature is increased, which is in agreement with our results. Additionally, as previously noted, higher extraction temperatures promote the weakening of carbohydrate-lignin bonds and thereby enhance the removal of carbohydrate [25]. With respect to carbohydrate removal the effect of extraction solvent pH was not significant compared to temperature [24]. Our results are in agreement as both the lignin samples isolated using pressurized 0.47 M NaOH at 180 °C and pressurized water at 180 °C were of comparable purity. However, it is noted that determining the neutral sugars content and acid insoluble lignin content of the lignin samples would provide definitive evidence of purity. These tests were not performed.

Table 3 presents the results of the nitrobenzene oxidation studies which were used to determine the yields and monomeric composition of the phenolic compounds present in the extracted lignin samples and unprocessed flax straw. The monomeric compositions are presented in terms of p-hydroxybezaldehyde (p-BA), vanillic acid (VA), acetovanillone (AVN), sryingic acid (SA), syringaldehyde (SAL), and ferulic acid (FA). Ferulic acid is produced from the base catalyzed hydrolysis of ester bonds between ferulic acids and guaiacyl-syringyl-type lignin. Table 3 shows that for all substrates, the dominant phenolic compound was vanillin followed by syringaldehyde. Predominant degradation products of vanillin and syringaldehyde result from the degradation of non-condensed guaiacyl and syringyl units, respectively [21]. Day *et al*. [5] determined the monomeric composition of lignin from inner and outer flax stem tissues with nitrobenzene oxidation; the values which ranged from approximately 7.5 to 362 μmol/g lignin, while the flax used in this work yielded a value of ∼1882 μmol/g lignin. The differences may be due to genetic and environmental differences in the flax samples [8]. The lignin obtained from conventional alkali treatment with 1.25 M NaOH, heated at 80 °C for 5 h and PLPW treatment with 0.47 M NaOH, heated at 180 °C for 82 min exhibited the lowest yields of nitrobenzene oxidation products, 296.0 and 615.6 μmol/g lignin, respectively, while the PLPW extracts obtained from the processing conditions of water-180°C, 0.47 M NaOH-100 °C and 0.47 M NaOH-140°C all demonstrated higher phenolic contents with 975.7, 898.6 and 867.5 μmol/g lignin, respectively. From the results presented inTable 3, it appears that the yields of all phenolic compounds obtained from alkaline assisted PLPW extraction decreased with temperature increasing from 100 to 180 °C, except the AVN yield increased. This result may be explained by the fact that alkaline hydrolysis allows for oxidative cleavage of lignin to aromatic aldehydes such as vanillin. The development of aceto-derivative by-products (such as acetovanillone, AVN) was likely enhanced at higher temperatures. Therefore a greater yield of acetovanillone was observed at the higher alkaline assisted PLPW extraction temperatures. Moreover, the results presented in Table 3 seem to contradict the results presented in Table 2 in that the 0.47 M NaOH-180 °C PLPW extraction condition yielded the highest lignin content. However, in nitrobenzene oxidation analysis, monomer identification is affected by the degree of condensation of the lignin, and the low yield of nitrobenzene oxidation products in these samples may be attributed to either the formation of condensed structures or the selective dissolution of condensed structures of lignin during the pressurized alkaline high temperature treatment [31,32]. On a lignin basis, all of the extracted lignin samples displayed lower phenolic contents, ranging from 296–975 μmol/g lignin compared to the unprocessed flax straw, ∼1882 μmol/g lignin. This result may indicate that the lignin samples all contained carbohydrate contamination to some degree; the extraction conditions induced condensation of the lignin molecules; or a combination thereof.

Table 3 also presents the relative total moles of p-hydroxybezaldehyde, represented by (H, hydroxyphenyl lignin units); the relative total moles of syringaldehyde, syringic acid, and acetosyringone, represented by (S, syringyl lignin units); and the relative total moles of vanillic acid, vanillin, acetovanillone represented by (G, guaiacyl lignin units) present in the flax shives lignins determined by the nitrobenzene oxidation. Day *et al*. [5] reported S/G ratios based on the monomeric composition of lignin as determined by alkaline nitrobenzene oxidation of inner and outer flax stem tissues, ranging from 0.14 to 0.43, which is in agreement with our results, ranging from 0.18–0.28. However, there appeared to be no discernable trend within our results. Based on the nitrobenzene oxidation data, the relatively low S/G ratio could mean that lignin fractions with more guaiacyl units were easier to extract than the lignin fractions with more syringyl units or that the guaiacyl units were less condensed or cross linked than the syringyl units [33,34].

### 2.2. Pyrolysis-GC-MS of Flax Shive Lignin

Pyrolysis-GC-MS was used to determine the compounds present in the lignin samples obtained from conventional alkaline extraction with 1.25 M NaOH at 80 °C for 5 h, alkali assisted PLPW extraction with 0.47 M NaOH at 180 °C, and the unprocessed flax shives. Table 4 shows the peak areas of the identified compounds. Key lignin derived compounds such as guaiacol, 4-vinyl guaiacol, 4-methyl guaiacol, syringol, eugenol, isoeugenol, catechol, homocatechol, and vanillin were detected in all of the samples. Other products such as furfural and levoglucosan were detected; these non-phenolics were derived from carbohydrates. The phenols, guaiacol, 4-methyl guaiacol, catechol, homocatechol, 4-vinyl guaiacol, syringol, and eugenol, and *trans*-isoeugenol were all concentrated to the greatest extent in the lignin obtained from extraction with PLPW. Phenols are valuable products with high commercial value as phenols are the starting material in the industrial production of aspirin, herbicides, and synthetic resins [1]. Lignin obtained from the conventional 1.25 M alkaline treatment yielded the lowest amounts of the key lignin derived compounds. Vanillin was present in both the lignin obtained from alkaline assisted pressurized water processing and the unprocessed flax shives in comparable amounts.

Overall, the molar yield of the guaiacyl (G) type lignin was about 4.7, 13.1, and 19.7 times higher than the syringyl (S) type lignin for the 1.25 M NaOH extracted lignin, alkaline assisted PLPW extracted lignin, and the unprocessed flax shives, respectively. Or in other words, the S/G ratio was 0.21, 0.08, and 0.05 for the 1.25 M NaOH extracted lignin, alkaline assisted PLPW extracted lignin, and the unprocessed flax shives, respectively. These values indicate a greater effect of extraction conditions on S/G ratio compared to the nitrobenzene oxidation data which indicated comparable S/G ratios for all samples. The pyrolysis data may be more reflective of the monomer composition of the samples since the main factor affecting release of monomer products during pyrolysis is the amount of energy required for the rupture of primary bonds (C-C or C-O-C) [35]. With nitrobenzene oxidation monomer, identification is affected by the degree of condensation of the lignin as it only determines the phenolic monomers present in uncondensed lignin. Also, nitrobenzene oxidation results are dependent on the choice (variety and identity) of external standards employed for chromatography based identification. Comparison of the nitrobenzene oxidation results with the pyrolysis GC-MS results indicates that the 1.25 M NaOH treatment extracted mainly uncondensed lignin as the S/G ratios for each method were similar. However, for the pyrolysis GC-MS obtained S/G ratio for the flax shives and alkaline assisted PLPW lignin were much lower compared to the nitrobenzene obtained S/G ratio indicating the presence of condensed lignin. The total yield of pyrolysis lignin products was 13.3, 64.7, and 30.5% for the 1.25 M NaOH extracted lignin, alkaline assisted PLPW extracted lignin, and the unprocessed flax shives, respectively. The work of Yang *et al*. [35] indicated that the total yield of pyrolysis lignin products for enzyme acid hydrolysis lignin was 52.8%; however it should be noted that during the pyrolysis process many chemicals further evolve into other compounds via secondary reactions. Curiously, the total lignin content of the flax shives, as determined with standard procedure [22], was 27.3%, which is comparable to the total yield of pyrolysis products (30.5%) obtained for the unprocessed flax shives. Levoglucosan was found in very large quantities (39.5%) in the 1.25 M NaOH, 80 °C, 5 h alkali extracted lignin, while less (2.5%) was detected in the alkaline PLPW extracted lignin. The presence of levoglucosan is likely due to carbohydrate contamination, as it is a 6 carbon ring structure formed from the pyrolysis of carbohydrates such as starch and cellulose. This is in agreement with the UV spectroscopy data as the presence of starch has been noted to decrease the UV absorbance of lignin [36]. Analytical pyrolysis proved to be a valuable technique in determining the chemicals that can be released upon exposure of biomass to high heat levels. This work shows the potential of pyrolysis as a method for obtaining high value chemicals from biomass. The effect of different variables such as temperature and heating time on the yield of chemicals produced will be investigated in future studies, allowing for a more detailed discussion on the behavior of different pyrolysis products.

## 3. Experimental Section

### 3.1. Samples

Flax shives were obtained from Biolin Research Inc. (Saskatoon, Canada) and ground using a Wiley mill (Arthur H. Thomas Co., Philadelphia, PA) using a 0.35 mm blade gap and a 4 mm screen. The screened flax shives with a particle size between 1 and 2 mm were further separated by air flotation to remove residual fiber. The ground flax shives were kept in sealed bags in a freezer at −25 °C.

### 3.2. Flax Shives Composition Determination

The lignin, cellulose, hemicellulose, ash, and wax content of flax shives were determined using published methods. The following method [20] was used to determine wax content. Briefly, flax shives were subjected to extraction with toluene and ethanol (2:1, v/v) in a Goldfisch apparatus for 5 h. Determination of the lignin was performed using the standard method published by the National Renewable Energy Laboratory [22]. Cellulose, and hemicellulose content of the flax shives was performed as described by Kim and Mazza [18]. For lignin content measurement, lignocellulosic material is subjected to a two step acid hydrolysis. The first hydrolysis step is performed using 72% H_2_SO_4_ followed by hydrolysis with 4% H_2_SO_4_ to solubilize all of the carbohydrate components. The resulting residue is termed acid insoluble lignin (or Klason lignin) and the low molecular fraction of lignin presenting the filtrate is called acid soluble lignin and quantified using UV spectroscopy [22]. The ash content of flax shives was determined by gravimetric analysis at 575 °C according to NREL standard procedure [39].

### 3.3. Lignin Extraction

Lignin was extracted from the samples using two different methods. The first step for both extraction methods involved drying the flax in an oven at 60 °C for 16 h. Method 1 was performed by following the alkaline hydrolysis procedure of Sun *et al*. [20] with some modifications. Briefly, as a first step, ground flax shives were dewaxed for 5 h using toluene and ethanol (2:1, v/v) using a Goldfisch apparatus. To obtain alkali soluble lignin, the dewaxed flax shives were subjected to hydrolysis with 1.25 M NaOH at 80 °C for 5 h (2 g sample per 44 mL extractant). The hydrolysate was filtered with a glass filter and washed with water (3 × 10 mL), followed by ethanol (2 × 10 mL, and acetone (1 × 10 mL). This created two fractions, a filtrate and an alkaline treated residue. The pH of the filtrate was reduced to 5.5 by addition of 6 N HCl. The hemicelluloses were separated from the filtrate by precipitation of the neutralized filtrate in 3 volumes of ethanol. After filtration with a glass filter, the precipitated material (*i.e.*, hemicellulose) was washed with 70% ethanol and allowed to air dry. Ethanol was evaporated from the filtrate, and the alkali soluble lignins were obtained from the filtrate by precipitation at pH 1.5 by addition of 6 N HCl. The alkali soluble lignin was washed with acidified water (pH 2), centrifuged and freeze dried. All extractions were conducted in triplicate.

Method 2 involved using PLPW (PLPW) processing to extract lignin from the flax shives. The extraction conditions were as described by Kim and Mazza [18] with the following modifications. Experiments were conducted using an extraction cell 40 cm length and 1.8 cm i.d. Dry flax shives (17 g), milled to a particle size range between 0.25 and 1 mm, were placed in the extraction cell. The extraction procedure was initiated by disconnecting the top fitting of the extraction cell and filling the cell with water or 0.47 M NaOH. The extraction temperature was 100, 140 or 180 °C, pressure was 5.2 MPa (750 psi), and the flow rate was 5 mL/min. Total processing time was 82 min. The lignin present in the flax shives extract after PLPW processing was isolated. The pH of the PLPW extract was reduced to 5.5 by addition of 6 N HCl. The hemicelluloses were separated from the PLPW extract by precipitation of the neutralized liquid in 3 volumes of ethanol. After filtration on a glass filter, ethanol was evaporated, and the PLPW soluble lignins were obtained by precipitation at pH 1.5 by addition of 6 N HCl. The PLPW extracted lignin was dried in a vacuum oven at 35 °C for 16 h. The total carbohydrates present in the extracts were estimated using the phenol-sulfuric acid method using standards of d-glucose [40,41]. All experiments were conducted in triplicate.

### 3.4. Nitrobenzene Oxidation

The composition of the noncondensed monomeric units of lignin was characterized by nitrobenzene oxidation. Following the methods [18,42], lignin samples (25 mg) were added to a mixture of 5 mL of 2 M NaOH and 0.5 mL of nitrobenzene and held in a pressurized tube reactor at 160 °C for 3 h. The reaction mixture was cooled down, and the solutions were extracted with 50 mL of diethyl ether three times and acidified to pH 1 with 6 N HCl. This solution was further extracted with 50 mL of diethyl ether three times. The extract was dried overnight (16 h) in a vacuum oven. The residue was dissolved in methanol. HPLC was used to determine the composition of the resulting phenolic aldehydes and acids.

### 3.5. HPLC Analysis of Phenolic Compounds

Analysis of phenolic compounds was conducted on an Agilent 1100 HPLC system with a G1329A autosampler and a G1312A pump, which was controlled by Agilent ChemstationPlus software (Agilent Technologies, Palo Alto, CA). The HPLC method used a Luna C18 (5 μm, 150 mm × 3.0 mm column) coupled with a C18 Security Guard cartridge, both from Phenomenex (Torrance, CA). The injector and column temperatures were set at 35 °C, and the injection volume was 20 μL. The mobile phases consisted of methanol (solvent A) and 4.4% (v/v) formic acid (solvent B) with a gradient as described by Kim and Mazza [28]. Briefly, the gradient consisted of 10% A at the start of the run, 25% A at 30 min run time, 35% A at 40 min run time, 60% A at 50 min run time, 100% A at 60 min run time, and 10% A at 70 min run time. The phenolic compounds were detected with a diode array detector at 280 nm. Phenolic compounds were identified and quantified by comparison with the retention times and the UV absorbance spectra of authentic standards obtained from Sigma Chemicals Co. (St. Louis, MO, USA).

### 3.6. UV Spectroscopy

UV spectra were recorded on a Cary 50 Bio UV-Visible Spectrophotometer (Varian, Palo Alto, CA). Isolated lignin sample was vacuum dried at 50 °C for 24 h. The dried lignin sample (5 mg) was dissolved in 10 mL of dioxane-water (95%, v/v). A 1 mL aliquot was diluted to 10 mL with 50% (v/v) dioxane-water, and the absorbance between 205 and 380 nm was measured [27].

### 3.7. Pyrolysis-GC-MS

For this work, the Pyrolysis-GC-MS method of Ralph and Hatfield [15] was followed. Samples, 200–300 μg were pyrolyzed in a quartz tube in a Pyroprob 5150 (CDS analytical Inc., Oxford, PA) at 700 °C for 10 s using helium as the carrier gas. The sample was carried onto a 30 m × 0.32 mm i.d. × 0.25 μm DB-1 (J&W Scientific) column fitted in an HP 5890 GC. The temperature was held at 50 °C for 10 min and then ramped at 4 °C/min to 290 °C. The eluting compounds were detected with an Agilent 5973 mass selective detector controlled by MSD chemstation (Agilent Technologies, Palo Alto, CA). Ionization was carried out at a 70 eV electron impact voltage in an ion chamber heated at 250 °C. The mass range scanned was m/z 70–600. Peak identifications were carried out on the basis of mass fragmentation patterns, and by comparing the MS data with NIST and Wiley Libraries.

## 4. Conclusions

Lignin extraction in terms of composition and yield was affected by the extraction method: solvent type, time and temperature were all important variables. Alkali assisted PLPW extraction was very effective in extracting lignin from flax shives. The amount of lignin present in the 0.47 M NaOH-180 °C extract was nearly 241 g kg^−1^ flax shive and the UV spectra of this lignin demonstrated its purity. With respect to the alkali assisted PLPW extract, temperature created an opposing effect on lignin yield and nitrobenzene oxidation products. The highest lignin yield was obtained from the 0.47 M NaOH-180 °C extraction condition; however this lignin presented the lowest nitrobenzene monomer yields, indicating a more condensed structure. Although the amount of lignin present in the PLPW extract obtained from processing with 180 °C water was less, 27 g kg^−1^ lignin, this lignin was also relatively pure and possessed a less condensed structure as shown by UV spectroscopy and nitrobenzene oxidation, respectively. This work also presents a detailed component analysis, including peak number, retention time, compound name, and relative proportion (% area) of the pyrolysis compounds of the lignin obtained by the different extraction methods using pyrolysis GC/MS. Overall, the majority of the products formed from the pyrolysis of lignins extracted by different methods were phenols with significant amounts of guaiacol, vanillin, and sryingol being detected. Comparison of the nitrobenzene oxidation results with the pyrolysis GC-MS results provided further insight into the degree of condensation exhibited by the different samples. In all, the results of this study demonstrate the potential of flax shives to serve as a source of lignin and consequently valuable biochemicals.

## Figures and Tables

**Table 1:** Composition of flax shives.

Component	Content (g kg^−1^ Flax Shives)
Cellulose	333 ± 5.0
Hemicellulose	219 ± 5.0
Acid Insoluble Lignin	260 ± 1.8
Acid Soluble Lignin	13 ± 0.1
Ash	15 ± 0.4
Wax	35 ± 2.4

Values are means ± standard deviations.

**Table 2:** Effect of extraction condition on lignin yield and composition of extracts.

Extraction Conditions	Lignin Yield a (g kg^−1^ Flax Shives)	Lignin Extraction Efficiency b (%)	Carbohydrate Content c (g kg^−1^ Flax Shives)	Free Phenolic Content d (g kg^−1^ Flax Shives)
Method 1—Alkali hydrolysis: 1.25 M NaOH, 80 °C, 5 h	92 ± 20	33.7	N/M	N/M
Method 2—PLPW: 0.47 M NaOH: 100 °C, 82 min	33 ± 2	12.1	177 ± 2	1.0 ± 0.1
Method 2—PLPW: 0.47 M NaOH: 140 °C, 82 min	72 ± 1	26.4	213 ± 2	3.0 ± 0.3
Method 2—PLPW: 0.47 M NaOH: 180 °C, 82 min	241 ± 32	88.3	302 ± 6	4.7 ± 0.3
Method 2—PLPW: Water: 180 °C, 82 min	27 ± 5	9.9	311 ± 2	3.9 ± 0.1

Values are means ± standard deviations.

a Lignins were obtained by precipitation at pH 1.5 by addition of 6 N HCl after isolation of the solubilized hemicelluloses;

b calculated as g lignin extracted per g lignin present in flax shives;

c Determined by phenol-sulfuric acid reaction method, expressed as glucose equivalent;

d Total soluble phenolic compounds in extract after removal of hemicelluloses and lignin, expressed as vanillin equivalent. N/M = Not measured.

**Table 3:** Yield and profiles of phenolic compounds of lignins from flax shives as determined by nitrobenzene oxidation.

Substrate	Extraction Conditions	Phenolic Compounds (μmoles/g lignin)								Molar Ratio	
		p-BA	VA	SA	VN	SAL	AVN	FA	Total	S/G	H/G
Flax Shives	Unprocessed	36.1 ± 2.5	110.1 ± 8.9	39.4 ± 1.0	1309.2 ± 65.8	358.2 ± 12.1	11.5 ± 0.1	17.5 ± 0.1	1882	0.28	0.025
Flax Shives Lignin^a^	1.25 M NaOH: 80 °C, 5 h	3.3 ± 0.8	29.8 ± 1.2	6.1 ± 1.5	194.7 ± 20.4	46.7 ± 5.5	10.8 ± 5.4	4.6 ± 1.1	296	0.22	0.014
Flax Shives PLPW Extract Lignin^b^	0.47 M NaOH: 100 °C, 82 min	14.8 ± 4.1	105.9 ± 14.9	28.3 ± 7.1	611.2 ± 44.1	138.5 ± 43.9	N/D	N/D	899	0.23	0.021
	0.47 M NaOH: 140 °C, 82 min	7.4 ± 0.8	82.1 ± 2.9	20.2 ± 1.0	594.7 ± 12.5	134.1 ± 12.6	18.7 ± 1.2	10.3 ± 0.5	868	0.22	0.011
	0.47 M NaOH: 180 °C, 82 min	4.1 ± 0.8	62.5 ± 9.5	14.1 ± 2.5	429.6 ± 98.1	78.1 ± 11.5	20.5 ± 3.6	6.7 ± 1.1	616	0.18	0.01
	Water: 180 °C, 82 min	8.2 ± 0.8	103.6 ± 1.8	25.3 ± 0.5	688.2 ± 19.7	150.6 ± 12.1	N/D	N/D	976	0.22	0.01

N/D: Not Detected. Abbreviations: p-BA, p-hydroxybenzaldehyde; VA, vanillic acid; SA, syringic acid; VN, vanillin; SAL, syringaldehyde; AVN, acetovanillone; FA, ferulic acid; H represents relative total moles of p-hydroxybezaldehyde; G represents relative total moles of vanillic acid, vanillin, and acetovanillone; S represents relative total moles of syringic acid, syringaldehyde. S/G = molar ratio of S to G lignin units; H/G = molar ratio of H to G lignin units.

a Nitrobenzene oxidation was performed with alkali soluble lignin. Lignin was obtained by precipitation at pH 1.5 by addition of 6 N HCl after isolation of the solubilized hemicelluloses;

b Nitrobenzene oxidation was performed with PLPW soluble lignin. Lignin was obtained by precipitation at pH 1.5 by addition of 6 N HCl after isolation of the solubilized hemicelluloses.

**Table 4:** Relative Proportion (% area) and Retention Times (RT) of the pyrolysis compounds in lignin obtained by different extraction methods.

Peak	RT (min)	Chemical Name	Origin	MW	Conventional Alkaline Extracted Lignin^a^	PLPW Extracted Lignin^b^	Flax Shives (FS)
1	1.51	Acetic acid	C	60	-	-	13.2
2	1.67	Tetrahydrofuran	C	72	-	-	-
3	1.85	Benzene	B	78	-	0.68	-
4	2.11	2,5-dimethylfuran	C	78	-	-	-
5	2.74	Toluene	C	92	0.38	1.41	0.85
6	3.04	3-Methylene-heptane	C	112	0.16	-	-
7	3.41	3-Furaldehyde	C	96	-	-	0.29
8	3.8	Furfural	C	96	-	-	2.65
9	4.4	3-Furanmethanol	C	98	-	-	0.79
10	6.26	2-Furanone	C	84	-	-	-
11	6.83	1,2-Cyclopentanedione	C	98	-	-	1.93
12	10.9	Phenol	H	94	0.50	1.91	-
13	13.3	Maple lactone	C	112	-	-	0.61
14	15.3	*o*-Cresol	H	108	0.30	1.05	0.59
15	16.4	*p*-Cresol	H	108	0.51	1.98	1.06
16	16.8	Guaiacol	G	124	1.29	8.61	1.85
17	19.8	2,4-Xylenol	H	122	-	0.38	-
18	20.9	*m*-Methylguaiacol	G	138	-	0.48	-
19	21.5	4-Methyl guaiacol	G	138	0.91	4.74	1.34
20	22.0	Catechol	L/Pp	110	1.22	5.15	2.89
21	22.8	Coumaran	C	120	-	0.56	-
22	23.0	5-Hydrxoymethylfurfural	C	126	-	-	1.26
23	24.1	3-Methoxy catechol	L/Pp	140	-	1.19	0.70
24	24.3	3-Methyl catechol	L/Pp	124	-	1.35	0.77
25	24.9	4-Ethyl guaiacol	G	152	0.29	1.64	-
26	25.4	Homocatechol	L/Pp	124	0.78	3.05	3.46
27	26.1	4-Vinyl guaiacol	G	150	1.49	8.24	2.53
28	27.4	Syringol	S	154	0.43	2.69	0.44
29	27.6	Eugenol	G	164	0.44	1.72	0.88
30	28.6	4-Ethylcatechol	L/Pp	138	-	1.18	-
31	28.9	Vanillin	G	152	0.95	2.82	2.61
32	29.3	*trans*-Isoeugenol	G	164	1.15	1.05	0.52
33	30.6	*cis*-Isoeugenol	G	164	-	6.69	2.70
34	30.8	4-Propylguaiacol	G	166	-	-	0.89
35	31.5	Levoglucosan	C	180	39.54	2.45	0.53
36	31.7	Acetovanillone	G	166	-	2.52	1.38
37	35.2	4-Allylsyringol	S	194	-	0.44	-
38	36.7	Syringaldehyde	S	182	-	0.42	0.54
39	37.2	*cis*-Coniferyl alcohol	G	180	1.31	0.84	0.63
40	38.0	Methoxy eugenol	G	194	-	1.39	0.54
41	38.7	4-Hydroxy-2 methoxycinnamaldehyde	H	178	-	0.92	2.13
42	38.8	*trans*-Coniferyl alcohol	G	180	-	2.26	2.0
43	45.0	*trans*-Sinapaldehyde	S	208	1.73	-	-

		SUM (L + H + G + S)			13.3	64.7	30.5

C = carbohydrate type structure; B = benzene type structure; L = non-specific lignin [37]; Pp = polyphenolic structure [38]; H = hydroxyphenyl type lignin; G = guaiacyl type lignin; S = syringyl type lignin; MW = molecular weight.

a Conventional Alkaline Extracted Lignin: Alkali lignin isolated from flax shives using 5% NaOH at 80 °C for 5 h;

b PLPW Extracted Lignin: Lignin in flax shives isolated with 0.47 M NaOH-180 °C PLPW extraction.

Flax shives: (FS).
